# Fermented Deer Blood Ameliorates Intense Exercise-Induced Fatigue via Modulating Small Intestine Microbiota and Metabolites in Mice

**DOI:** 10.3390/nu13051543

**Published:** 2021-05-03

**Authors:** Jingwen Cui, Chao Shi, Peibin Xia, Ke Ning, Hongyu Xiang, Qiuhong Xie

**Affiliations:** 1 Key Laboratory for Molecular Enzymology and Engineering of Ministry of Education, School of Life Sciences, Jilin University, Changchun 130012, China; cuijw17@mails.jlu.edu.cn (J.C.); shichao19@mails.jlu.edu.cn (C.S.); xiapb17@mails.jlu.edu.cn (P.X.); ningke20@mails.jlu.edu.cn (K.N.); qhxie@jlu.edu.cn (Q.X.); 2 National Engineering Laboratory for AIDS Vaccine, School of Life Sciences, Jilin University, Changchun 130012, China; 3 School of Life Sciences, Jilin University, Changchun 130012, China; 4 Fusong Institute of Changbai Mountain Resource and Health, Fusong 134504, China

**Keywords:** exercise-induced fatigue, fermented deer blood, antioxidant, small intestinal microbiota, metabolomics, intestinal health

## Abstract

Intense and excessive exercise-induced fatigue has become an important health issue and can damage intestinal health. Deer blood, as a food byproduct with nutritional value, has been found to restore physical strength. However, little is known about the antifatigue effect of fermented deer blood (FDB) on intense exercise mice. The purpose of the present study is to investigate the antifatigue effect of FDB, and whether this effect is correlated with the altered small intestinal microbiota and metabolites in exercise mice. In this study, 5-week-old male C57BL/6J mice are given treadmill exercise with or without FDB supplementation (30 and 150 mg/kg/d) for 3 weeks. FDB significantly reduces metabolic byproduct accumulation, liver and intestinal damage, and enhances glycogen storage and antioxidant capacity in intense exercise mice. Moreover, FDB restructures the small intestinal microbiota by increasing the abundance of probiotics and butyric acid producing bacteria and decreasing the abundance of pathogenic bacteria. FDB also regulates the levels of metabolites involved in TCA cycle and amino acid metabolism in urine and small intestine content. Correlation analysis shows that FDB-modulated microbiota is highly associated with its antifatigue effect. FDB may ameliorate fatigue and intestinal injury through targeting small intestinal microbiota.

## 1. Introduction

With the improvement of living standards, more and more people are concerned about healthy lifestyles. It is well known that exercise is popular for its many health-related benefits. Physical exercise has a positive impact in reducing and managing the risk of a range of chronic diseases, mental health, and life expectancy [1]. However, intense exercise and excessive exercise can produce reactive free radicals and increase accumulated metabolites, leading to physical fatigue [2,3]. Many studies have confirmed that exercise-induced fatigue can induce intestinal barrier damage and changes in intestinal microbial composition in mice [4,5]. Additionally, the altered composition of the gut microbiota has been reported to cause chronic fatigue syndrome or myalgic encephalomyelitis (CFS or ME) [6,7,8]. Therefore, the development of therapeutic measures that can restore intestinal barrier function, and regulate intestinal microbiota is the key to ameliorate fatigue. Several chronic diseases are related to changes in microbiota composition, such as fatigue, obesity, diabetes, and inflammatory bowel disease [9,10]. To date, there has been growing interest in understanding the interaction between exercise fatigue and gut microbiota. Most studies have relied on examining the cecal or fecal microbiota. The small intestine is essential for macronutrient absorption and energy acquisition [11]. A critical and unanswered question is whether intense exercise has a significant impact on the small intestinal microbiota, which has profound effects on various aspects of host physiology, including immune, metabolic, and endocrine functions [12,13].

Recently, a variety of anti-fatigue nutritional beverages have appeared on the market, among which the most common components are caffeine, taurine, and guarana [14]. However, there are serious concerns about the safety of these beverages. Therefore, there is an urgent need to develop effective functional products to improve exercise capacity and relieve fatigue without side effects. Many studies have found that supplements of protein, sugars, peptides, and amino acids can reduce the accumulations of harmful metabolites, improve antioxidant levels and relieve fatigue [4,5,15,16,17]. Among them, protein hydrolysates and peptides are popular because of their high activity, easy absorption, and low toxicity. Blood is a protein-rich meat processing byproduct. Tons of blood are collected in slaughterhouses each year, which are either processed into blood powder or discarded as effluent [18,19]. Blood is also used in traditional Asian and European foods for its rich protein and nutritional value, such as blood sausages and blood tofu [20]. The food processing of animal blood is a challenge to improve its nutritional and commercial value. Fermentation is an effective way to process and preserve animal blood. In recent years, fermented foods are experiencing increased interest. Fermentation has been found to improve the nutrient content, flavor and function of food. *Bacillus* spp. and LAB are widely used for fermented products, due to their ability to produce various hydrolytic enzymes to hydrolyze macromolecules and produce small molecular substances with physiological activity, such as active peptides and amino acids [14]. It has been shown that animal substances can be fermented with probiotics to effectively enrich active components and improve some physiological functions (antioxidant, antimicrobial, and antihypertensive activity, etc.) [21,22,23].

Deer blood is a typical animal blood, and it is also a functional food in China. Proteins and polypeptides are considered to be the most prominent bioactive components of deer blood and they possess a variety of physiological functions, such as anti-aging, anti-fatigue, enhancing immunity, enriching blood, and calming nerves. Over the years, deer blood was only consumed as deer blood wine and deer blood powder products in Asia. However, few studies have used probiotics to ferment deer blood to enrich active components and improve its antioxidant and antifatigue activities. Furthermore, the mechanism by which fermented deer blood products relieve fatigue is not yet clear.

The purpose of this study was to investigate the beneficial effects of fermented deer blood (FDB) on intense exercise-induced fatigue by detecting metabolic byproduct accumulation, antioxidant capacity, intestinal barrier function, gut microbiota, and metabolites. Furthermore, the correlation analysis of these effects was also performed to investigate the association between small intestinal microbiota and fatigue related parameters in mice.

## 2. Materials and Methods

### 2.1. Strains and Medium

*Bacillus subtilis* 22, *Lactobacillus rhamnosus* CY-4, and *Enterococcus faecium* GG-4 were isolated from fermented soybean food and identified by 16S rDNA similarity analysis. *Lactobacillus plantarum* subsp. *plantarum* CICC 20315 and *Lactobacillus paracasei* CICC 20288 were purchased from the China Center of Industrial Culture Collection (CICC). These strains were stored at −80 °C. Prior to fermentation, *Bacillus* and LAB strains were inoculated into 50 mL of LB and MRS medium, respectively, until the cell densities were greater than 10^8^ CFU/mL. The bacterial suspensions were used for deer blood fermentation.

### 2.2. Preparation of Fermented Deer Blood

The deer blood powder was provided by Jilin Guolu Biological Technology Co., Ltd. (Changchun, China). The company is specialized in sika deer breeding and sales of deer products. The deer blood fermentation was performed as shown in Figure S1. Deer blood powder was mixed with 20 times the weight of water and then sterilized at 121 °C for 20 min to obtain a sterile deer blood solution. Then, 2% inoculum of the *B. subtilis* 22 was added to sterile deer blood solution (5%, *v/v*), and incubated at 37 °C for 96 h. Next, the shaker temperature was raised to 50 °C for 8 h, and when the temperature returned to 37 °C, four LAB strains (*L. rhamnosus* CY-4, *E. faecium* GG-4, *L. plantarum* CICC 20315, and *L. paracasei* CICC 20288) were separately inoculated into the substrate and incubated at 37 °C for 24 h. Next, the fermentation broth was centrifuged at 4000 rpm, and the supernatant and precipitate were collected. The supernatant was concentrated (40 to 50% solid) and then mixed with the precipitate. Finally, the mixture was lyophilized and powdered as a fermented deer blood product (FDB) for further analysis.

### 2.3. Peptide and Amino Acid Content of Fermented Deer Blood

The peptide content was determined by the modified bicinchoninic acid method [24]. Amino acids were quantitatively analyzed by automatic amino acid analyzer (S433D, SYKAM, Eresing, Germany). Deer blood samples were suspended in distilled water and treated with ultrasound for 30 min. Then, 500 µL of 10% sulfosalicylic acid solution was added to a 500 µL aliquot of the sample. Next, the sample was centrifuged at 14,000 rpm for 10 min, and the supernatant was collected to detect amino acids.

### 2.4. Antioxidant Activity Assay of Fermented Deer Blood

The analysis of DPPH radical, superoxide radical and hydroxyl radical scavenging activity, and ferric reducing antioxidant power (FRAP) referred to the published methods [15,25,26].

### 2.5. Animal Experimental Design

All animals were maintained in accordance with the laboratory animal guidelines, and the animal experiment was approved by the Institutional Animal Care and Use Committee of Jilin University (Permission Number: 2018-SY1206). In total, 50 male C57BL/6J mice (five-week-old) were obtained from Vital River Laboratory Animal Technology Co., Ltd. (Beijing, China). After one-week acclimatization, the mice that could not keep up with the treadmill at 20 m/min were excluded. Then 40 mice were randomly assigned to four groups: normal control (NC), exercise control (EC), 30 mg/kg FDB (FDB30), 150 mg/kg FDB (FDB150). The schematic diagram of animal experiment illustrating the exercise protocol and feeding strategies is shown in Figure S2. The FDB groups received FDB (30 and 150 mg/kg) once a day for three weeks. The NC and EC groups were given equal volumes of distilled water. Except for the NC group, the other mice exercised on a treadmill every other day. The exercise program was performed using the following protocol: running at 15 m/min for 3 min to warm up, and then increasing the speed 2 m/min every minute until 21 m/min. After that, the speed was increased at a rate of 1 m/min every 2 min. Mice were considered exhausted when they refused to run even if they were electrically stimulated [27]. On the last day, all mice rested for 30 min after running and were sacrificed.

### 2.6. Serum and Liver Biochemical Analysis

Biochemical analyses and antioxidant enzyme activity of serum and liver samples were performed as previously described [14], including hepatic glycogen (HG), urea nitrogen (BUN), lactic acid (LA), glucose, lactate dehydrogenase (LDH), aspartate aminotransferase (AST), alanine aminotransferase (ALT), superoxide dismutase (SOD), glutathione peroxidase (GSH-Px), total antioxidative capacity (T-AOC), and malondialdehyde (MDA). Serum lipopolysaccharide (LPS) was detected by the enzyme-linked immunosorbent assay (ELISA) kit (Cusabio Biotechnology, Wuhan, China) according to manufacturer’s instruction.

### 2.7. RNA Extraction and Quantitative Real-Time PCR (qRT-PCR)

The total RNA extraction, reverse transcription and qRT-PCR process are performed as described previously [14]. Primer sequences were presented in Table S1. GAPDH and 28S were used as the reference genes in the liver and ileum, respectively.

### 2.8. Gut Microbiota Analysis of Small Intestine and Cecal Contents

The bacterial DNA was isolated from small intestine and cecal contents and prepared for 16S rRNA gene sequencing analysis. Detailed steps of 16S rRNA gene V3-V4 region sequencing and data processing were referred to previous report [28]. The V3-V4 region of the 16S rRNA gene was amplified with a forward primer (5′-CCTACGGRRBGCASCAGKVRVGAAT-3′) and a reverse primer (5′-GGACTACNVGGGTWTCTAATCC-3′).

### 2.9. Metabolomic Analysis of Urine and Intestinal Content

The sample treatment method, NMR data acquisition and processing were performed using previously described methods [29]. The raw data were preprocessed using MestReNova 10.0. Multivariate analysis was performed using software SIMCA 14.0 (Umetrics, Umeå, Sweden), including principal component analysis (PCA) and orthogonal partial least squares-discriminant analysis (OPLS-DA). In OPLS-DA, potential biomarkers that had a significant contribution to group separation were identified, based on a variable importance on projection (VIP) value > 2 and *p* < 0.05. Metabolomics pathway analysis (MetPA) was performed using MetaboAnalyst.

### 2.10. Statistical Analysis

All data were presented as means ± SD. Statistical differences were determined by ANOVA analysis and Tukey’s test. Data were analyzed using SPSS 24.0 (SPSS Inc., Chicago, IL, USA) and performed with GraphPad Prism version 5.01 (GraphPad software, San Diego, CA, USA). The correlations were analyzed by Spearman’s correlation. Heatmaps were made using a Heml package (Heatmap Illustrator, version 1.0). A *p*-value of < 0.05 indicated a significant difference.

## 3. Results

### 3.1. Compositions and Antioxidant Activity of Fermented Deer Blood

Peptides and amino acids are the main functional components in fermented products. In this study, compared with non-fermented deer blood (DB), fermented deer blood (FDB) had higher peptide and free amino acid contents, reflecting the hydrolysis of deer blood protein during the fermentation process. The peptide content was 184.3 mg/g in the FDB (Table S2). In total, 139.7 mg/g amino acids were detected in the FDB (Table S3). Compared to the DB, a higher antioxidant activity was found for FDB. The increase in antioxidant activity may be due to the effects of antioxidant peptides and amino acids. The antioxidant capacities of FDB were evaluated with a combination of FRAP (0.76 mg FE/g), DPPH (14.86%), hydroxyl radical (62.89%), and superoxide (41.69%) scavenging activity (Table S2). In summary, FDB was rich in peptides and amino acids, and showed antioxidant activity.

### 3.2. FDB Alleviated Intense Exercise-Induced Fatigue and Oxidative Stress Response

**Figure 1:**
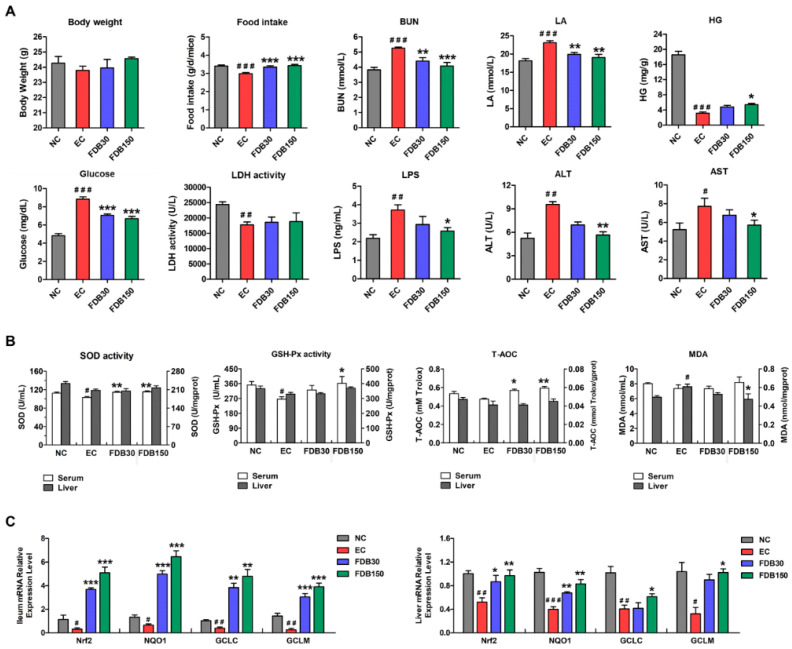
Effects of FDB on ameliorating fatigue and oxidative stress in intense exercise mice. (**A**) Serum biochemical parameters and HG. (**B**) SOD, GSH-Px, T-AOC, and MDA levels in the serum and liver. (**C**) The mRNA expression of oxidative stress-related signaling genes in the ileum and liver. ^#^ *p* < 0.05, ^# #^ *p* < 0.01, and ^# # #^ *p* < 0.001 vs. NC (normal control) group; * *p* < 0.05, ** *p* < 0.01, and *** *p* < 0.001 vs. EC (exercise control) group. FDB30: 30 mg/kg FDB group; FDB150: 150 mg/kg FDB group. BUN: urea nitrogen; LA: lactic acid; HG: hepatic glycogen; LDH: lactate dehydrogenase; LPS: lipopolysaccharide; ALT: alanine aminotransferase; AST: aspartate aminotransferase; SOD: superoxide dismutase; GSH-Px: glutathione peroxidase; T-AOC: total antioxidative capacity; MDA: malondialdehyde.

As shown in Figure 1A, the levels of serum BUN, LA, glucose, LPS, ALT, and AST were significantly increased in the EC group. In addition, food intake and HG were significantly reduced by intense exercise. FDB intervention obviously attenuated these effects. In EC and FDB groups, no significant difference was observed in the body weight. An imbalance between the body’s oxidation and antioxidant systems will lead to the progression of fatigue. Compared with NC group, intense exercise increased the MDA content in the liver that was significantly downregulated by the FDB150 treatment (Figure 1B). After FDB treatments, the levels of serum T-AOC, SOD, and GSH-Px activity were higher than those of the EC group. In addition, we evaluated the expression of oxidative stress-related signaling genes in the liver and ileum (Figure 1C). Our results showed that FDB significantly increased the expression levels of Nrf2, NQO1, GCLC, and GCLM mRNA when compared with EC group. FDB150 group showed the highest expression levels of these genes.

### 3.3. FDB Improved Intestinal Integrity and Inflammatory Response

**Figure 2:**
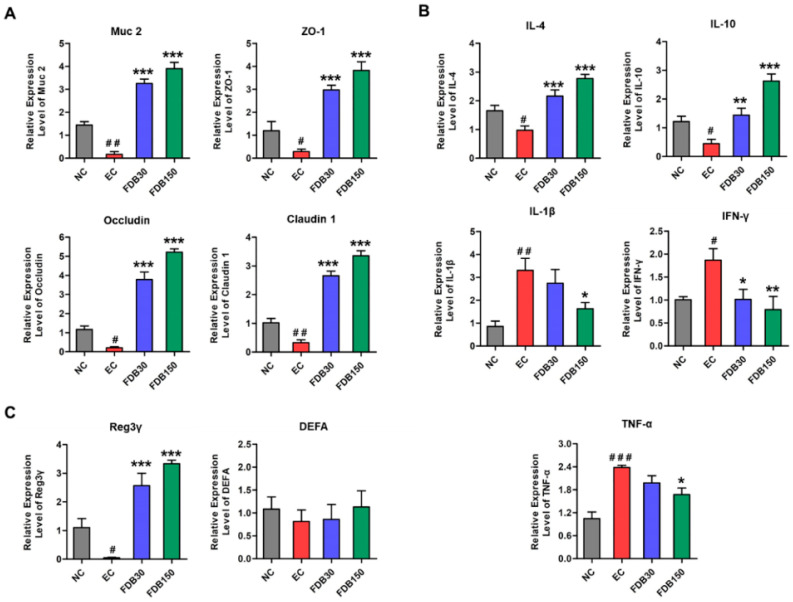
Effects of FDB on the mRNA expression of intestinal barrier protein, inflammatory cytokines, and antimicrobial peptide in the ileum. (**A**) Muc-2, ZO-1, Occludin, and Claudin 1; (**B**) IL-4, IL-10, IL-1β, IFN-γ, and TNF-α; (**C**) Reg3γ and DEFA. ^#^ *p* < 0.05, ^# #^ *p* < 0.01, and ^# # #^ *p* < 0.001 vs. normal control (NC) group; * *p* < 0.05, ** *p* < 0.01, and *** *p* < 0.001 vs. exercise control (EC) group. FDB30: 30 mg/kg FDB group; FDB150: 150 mg/kg FDB group.

The effects of FDB on the expression of genes involved in intestinal integrity, antimicrobial peptides and inflammation in ileum at mRNA level are shown in Figure 2A–C. Compared with the EC group, FDB significantly promoted the expression of Muc 2, ZO-1, Occludin, Caludin-1, Reg3γ, IL-4, and IL-10, and inhibited the expression of TNF-α, IL-1β, and IFN-γ. FDB150 showed better regulating ability than FDB30.

### 3.4. FDB Modulated the Gut Microbiota of Fatigued Mice under Intense Exercise

**Figure 3:**
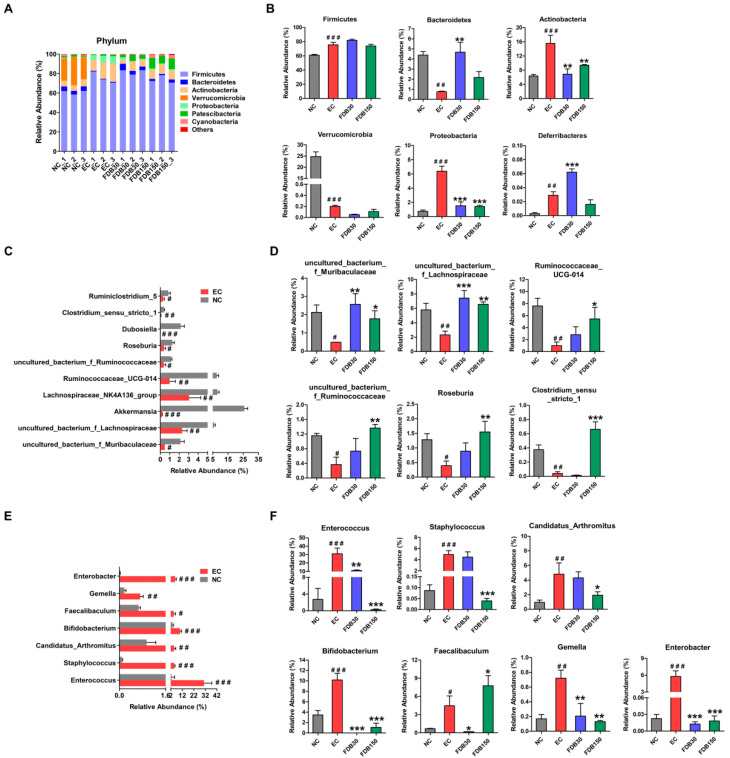
FDB supplementation altered the composition of small intestinal microbiota in fatigued mice. (**A**) Taxonomic distributions of bacteria from small intestine content 16S rDNA sequencing data at the phylum level. Relative abundance of significantly altered bacterial taxa at (**B**) phylum and (**C**,**E**) genus levels between NC and EC mice. (**D**,**F**) Relative abundance of significantly altered bacterial taxa at genus level between EC and FDB mice. ^#^ *p* < 0.05, ^# #^ *p* < 0.01, and ^# # #^ *p* < 0.001 vs. normal control (NC) group; * *p* < 0.05, ** *p* < 0.01, and *** *p* < 0.001 vs. exercise control (EC) group. FDB30: 30 mg/kg FDB group; FDB150: 150 mg/kg FDB group.

16S rRNA gene sequencing was performed to analyze the small intestine and cecum microbiota diversity and composition. Figure S3A shows that a significant increase in both diversity and richness of small intestinal microorganisms after FDB treatments. In terms of bacterial composition at phylum level (Figure 3A,B), FDB intervention could reverse the intense exercise-induced increases in the relative abundances of Actinobacteria and Proteobacteria. The changes in the microbiota at the genus level are shown in Figure S3B and Figure 3C–F. Among these genera, *Ruminococcaceae_UCG-014*, *Clostridium_sensu_stricto_1*, *Roseburia*, *uncultured_bacterium_f_Muribaculaceae*, *uncultured_bacterium_f_Lachnospiraceae,* and *uncultured_bacterium_f_Ruminococcaceae* were significantly downregulated in the EC group compared to the NC group (Figure 3C). In contrast, *Enterococcus*, *Staphylococcus*, *Candidatus_Arthromitus*, *Bifidobacterium*, *Faecalibaculum*, *Gemella,* and *Enterobacter* were significantly upregulated in the EC group (Figure 3E). FDB significantly regulated the relative abundance of these genera in mice under intense exercise (Figure 3D,F). Furthermore, FDB150 showed the strongest regulatory effect. Gut microbiome also was determined by sequencing using cecal content samples of mice (Figure S4A–D). There were no significant differences between the NC and EC group in diversity and richness (Figure S4A). Consistent with previous report, intense exercise influenced the relative abundances of cecal microorganisms in the mice. Furthermore, we found that FDB treatment reversed the relative abundances of Firmicutes, Bacteroidetes, Verrucomicrobia, *Bifidobacterium*, *uncultured_bacterium_f_Muribaculaceae*, *uncultured_bacterium_f_Erysipelotrichaceae*, *Faecalibaculum,* and *Lactobacillus* compared with EC group (Figure S4B–D).

### 3.5. Effects of FDB on the Metabolome of Urine and Intestine

**Figure 4:**
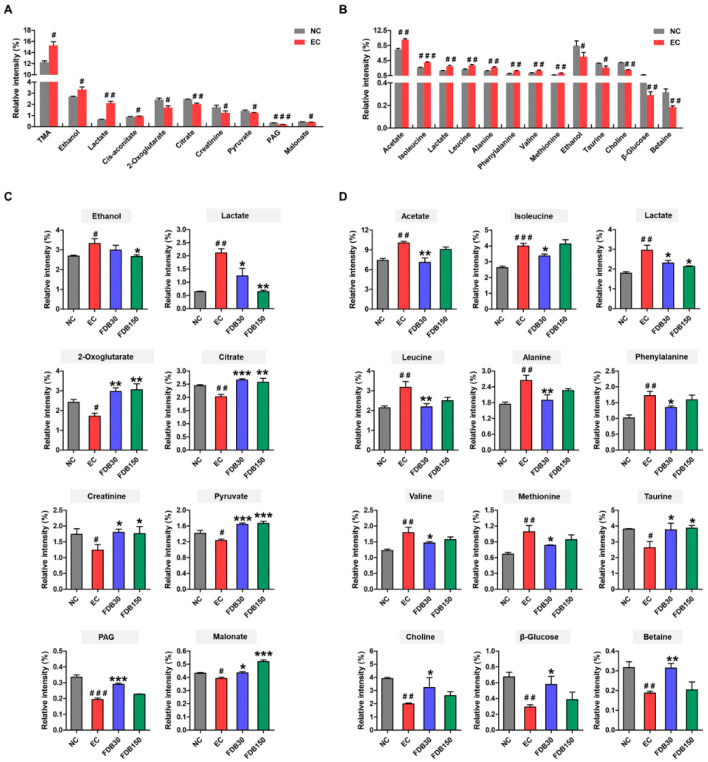
Effects of FDB on urinary and small intestine metabolites in fatigued mice. Significant changes in (**A**) urinary and (**B**) small intestine metabolites in NC and EC groups are shown in the histogram. Significant changes in (**C**) urinary and (**D**) small intestine metabolites in EC and FDB-treated groups are shown in the histogram. ^#^ *p* < 0.05, ^# #^ *p* < 0.01, and ^# # #^ *p* < 0.001 vs. normal control (NC) group; * *p* < 0.05, ** *p* < 0.01, and *** *p* < 0.001 vs. exercise control (EC) group. FDB30: 30 mg/kg FDB group; FDB150: 150 mg/kg FDB group. TMA: trimethylamine; PAG: phenylacetyl glycine.

NMR was performed to investigate urine and small intestine content samples from the four groups. OPLS-DA of samples demonstrated the separation of the NC and EC groups (Figure S5A,B). The S-plot and VIP were used to select potential biomarkers. The variables far from the origin and VIP > 2 could be considered as potential biomarkers related to exercise-induced fatigue (Figure 4A,B). Based on the metabolites significantly affected by intense exercise, the potential target metabolic pathways (impact > 0.10, −log (*p*) > 2) were identified by MetaboAnalyst.

Compared with EC group, FDB increased urinary metabolites 2-oxoglutarate, citrate, creatinine, pyruvate, phenylacetyl glycine (PAG), and malonate while decreasing ethanol and lactate compared to the EC group (Figure 4C). Pyruvate metabolism, TCA cycle, and glycolysis and gluconeogenesis were recognized as the key metabolic pathways in the formation of intense exercise-induced fatigue (Figure S5E). As shown in Figure 4B, the levels of 13 small intestine metabolites in the EC group were different from those in the NC group. The top seven metabolic pathways included taurine and hypotaurine metabolism, pyruvate metabolism, amino acid metabolism, and biosynthesis (Figure S5F). We observed that FDB lowered 6 amino acids including the branched chain amino acids (BCAA) valine, isoleucine, and leucine in the small intestine content. Compared with EC group, FDB increased taurine, choline, β-glucose, and betaine, but decreased acetate and lactate (Figure 4C). As shown in Figure S6A–E, cecal metabolites results indicated that FDB only significantly reduced the acetate, lysine, and isoleucine.

### 3.6. Association of Gut Microbial Dysbiosis with Fatigue Related Parameters and Dysregulation Metabolites

**Figure 5:**
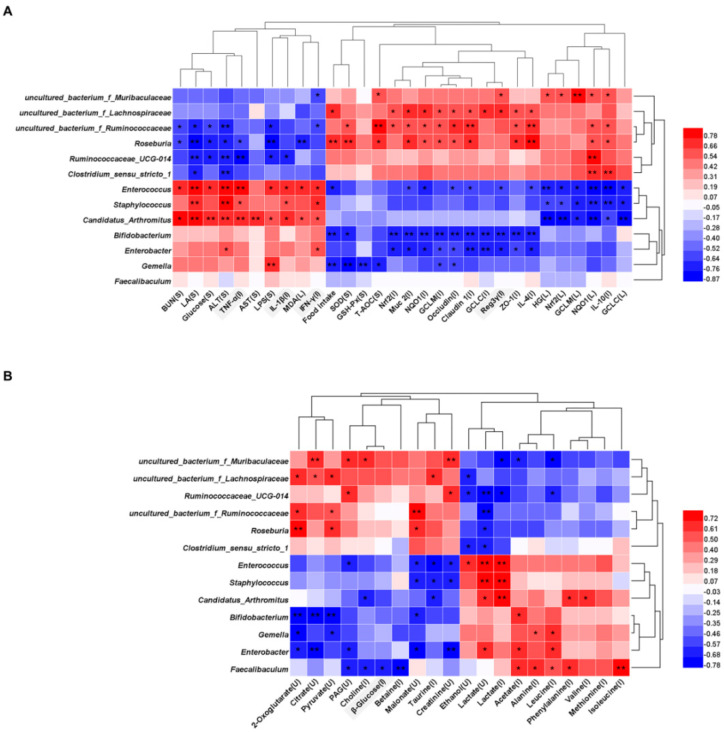
Correlation analysis between small intestine microbiota (genus level) reversed by FDB intervention with fatigue related parameters and metabolites. Correlation of 13 genera with (**A**) fatigue related parameters and (**B**) urinary and small intestine metabolites. The heat map represents the correlation coefficient value. The parameters and metabolites for the serum, liver, ileum and urine were labeled as S, L, I, and U, respectively. Significant correlations are marked by * *p* < 0.05, ** *p* < 0.01.

Spearman’s correlation analysis was performed to analyze the correlation between the 13 genera in small intestine microbiota reversed by FDB supplementation and fatigue related parameters in all the mice groups (Figure 5A). It was found that 12 genera were strongly correlated with a variety of indicators. For instance, these increased genera in the FDB treated groups included *Roseburia* and *uncultured_bacterium_f_Ruminococcaceae*, which were significantly positively correlated with SOD, T-AOC, antioxidant-related genes (Nrf2, NQO1, GCLC, and GCLM), intestinal barrier-related proteins (Muc 2, ZO-1, Occludin, and Claudin 1), anti-inflammatory cytokine (IL-10 and IL-4), and were negatively correlated with BUN, LA, Glucose, ALT, and LPS. *Ruminococcaceae*_UCG-014 showed a significant negative correlation with LA, Glucose, ALT, LPS, TNF-α, and IL-1β. *Enterococcus*, *Staphylococcus,* and *Candidatus_Arthromitus*, which were decreased after FDB supplementation, were significantly positively correlated with LA, ALT, TNF-α, IL-1β, and IFN-γ, and were negatively correlated with HG, IL-10, and antioxidant-related genes. *Enterobacter* and *Bifidobacterium* showed a significant negative correlation with antioxidant-related genes and intestinal barrier-related proteins in the ileum.

To further investigate the potential associations between altered small intestine microbiota and altered metabolites in urine and small intestine, we performed a Spearman’s correlation analysis (Figure 5B). Many genera were related to lactate, creatinine, 2-oxoglutarate, pyruvate, and citrate, implying a correlation with energy metabolism. *Faecalibaculum*, *Enterobacter*, *Enterococcus,* and *uncultured_bacterium_f_Muribaculaceae* were most correlated with metabolites. Malonate, PAG, 2-oxoglutarate and citrate, which obviously increased after FDB supplementation, showed a positive correlation with *Roseburia*, *uncultured_bacterium_f_Lachnospiraceae,* and *uncultured_bacterium_f_Ruminococcaceae*, and a negative correlation with *Bifidobacterium* and *Enterobacter*. Reduced acetate and amino acids showed a significant positive correlation with *Candidatus_Arthromitus*, *Bifidobacterium*, *Gemella*, *Enterobacter,* and *Faecalibaculum* in FDB mice.

## 4. Discussion

To evaluate the anti-fatigue effect of FDB, we determined relevant biochemical indices. When the body exercises excessively or vigorously, glycogen will be broken down to meet a need for glucose to supply energy. Fatigue will occur when stored liver and muscle glycogen are depleted [30]. During intense exercise, anaerobic metabolism will produce LA, and its accumulation leads to low metabolic efficiency. BUN is one of the main indicators to assess fatigue. The accumulation of LA and BUN is one of the important causes of fatigue [3,4,5]. The results suggested that FDB could significantly improve glycogen storage and blood glucose balance, and reduced the accumulation of LA and BUN. Similarly, the *Hippocampus* peptide has been reported to have anti-fatigue effects by reducing blood glucose, LA and BUN levels, and increasing hepatic glycogen storage [15]. The activity of ALT and AST in serum can predict the liver cell damage and exercise intensity [31]. FDB supplementation could ameliorate the liver injury in intense exercise mice. There is accumulating evidence that oxidative stress contributes strongly to fatigue. In this work, the in vitro results showed that FDB had high antioxidant activity. Meanwhile, FDB could improve the body’s antioxidant capacity by decreasing the level of MDA, and enhancing antioxidant enzyme activity. Moreover, FDB also regulated the expression levels of Nrf2 signaling-related genes in the ileum and liver of exercise mice. Nuclear factor E2-related factor 2 (Nrf2) can recognize and binds antioxidant response elements (ARE) and activates the transcription and translation of downstream antioxidant protein genes (NQO1, GCLC, and GCLM) [14]. The research of Duan et al. indicated that the potential mechanism of the anti-fatigue effect of luteolin-6-C-neohesperidoside is mainly through the activation of the Nrf2/ARE pathway to relieve oxidative damage [32]. In this study, FDB alleviated the intense exercise-induced oxidative response, possibly via activation of Nrf2/ARE antioxidant pathways. The results indicated that FDB could affect parameters related to fatigue and oxidative stress.

High intensity or strenuous exercise disrupts small intestinal barrier function and immune homeostasis, thereby increasing circulating bacteria and leading to systemic inflammation [4,33]. The physical barrier function of the intestinal mucosa was maintained by the tight junction between intestinal epithelial cells [34]. Claudin 1, Occludin, and ZO-1 are key markers of tight junctions. Mucin and antimicrobial peptides (AMP) form the chemical barrier of the intestinal mucosa that enhances intestinal protection [35]. Muc 2 is the main component of the mucin family [36]. DEFA and Reg3γ are typical antimicrobial peptides in the intestinal mucosal defense system. The reduced mRNA expression of these proteins increases intestinal epithelial permeability, leading to bacterial translocation and elevated LPS level. LPS is known to be a component of the cell walls of gram-negative bacteria that stimulates the epithelium of the digestive tract to produce inflammation [37]. In the present study, the enhanced expression of Claundin 1, Occludin, ZO-1, Muc 2, and Reg3γ in ileum of FDB-treated groups indicated FDB could improve intestinal barrier function, as supported by the result of down-regulated LPS level in serum.

In the intestinal immune system, mucosal cytokines play a key role in regulating intestinal barrier function and intercellular communication [38]. Some studies have suggested that a reduction in tissue inflammation may alleviate chronic fatigue, such as TNF-α, IL-1β, and IFN-γ play a major role in the development of chronic fatigue syndrome. In the ileum, FDB supplementation not only decreased the production of pro-inflammatory cytokines (i.e., IL-1β, TNF-α, and IFN-γ), but also markedly up-regulated the levels of anti-inflammatory cytokine (i.e., IL-10 and IL-4). Our study suggested that FDB intervention could protect the small intestinal physical, biochemical, and immune barriers from intense exercise-induced damage.

Many studies have found changes in the gut microbiota of subjects with CFS. In addition, excessive exercise could alter the diversity and the abundance of gut microbiota [5,39]. In our study, intense exercise had a major influence on the diversity and composition of small intestinal microbiota, including the increased pathogens and decreased butyrate-producing bacteria and probiotics. Recent studies have suggested that the increase in Proteobacteria is a potential marker for dysbiosis and fatigue related disease risks [40,41]. Proteobacteria are more abundant in patients with ME or CFS and inflammatory bowel disease (IBD) [42,43]. In this study, FDB supplementation significantly reduced Proteobacteria abundance in intense exercise mice. A recent study revealed that *Enterococcus* spp. in the fecal samples from the CFS group was significantly higher than the control group [44]. The relative abundance of pathogens, such as *Enterococcus* and *Enterobacter*, were decreased in the FDB-treated groups compared to the EC group. Recently, some studies have found that the abundance of the genus *Faecalibacterium* is significantly lower in the ME or CFS population, which has anti-inflammatory properties both in vitro and in vivo [45,46]. However, we observed that the relative abundance of *Faecalibacterium* was increased in the EC group when compared to the NC group. These findings indicated that the mouse strains, type of exercise and sample source may influence the diversity and abundances of gut microbiota. Some studies demonstrated that moderate exercise significantly increased butyrate-producing bacteria in fecal and cecal samples, such as *Clostridiaceae*, *Lachnospiraceae,* and *Ruminococcaceae* [47,48,49]. However, this study found that intense exercise caused a decrease in the abundance of these butyric acid producing bacteria. *Roseburia* regulated oxidative damage and inflammation in obese mice [50]. It is found that *uncultured_bacterium_ f_Muribaculaceae* is a beneficial bacteria in the research of various diseases. Our results showed that FDB supplementation increased the relative abundance of *uncultured_bacterium_f_Lachnospiraceae*, *Roseburia uncultured_bacterium_f_Muribaculaceae*, *uncultured_bacterium_f_Ruminococcaceae,* and *Ruminococcaceae_UCG-014* which was accompanied by the reduced abundance of *Enterococcus* and *Enterobacter*. In addition, the results of correlation analysis suggested that the anti-fatigue effect of FDB was related to the change of small intestine microbiota. Thus, these butyrate-producing bacteria and probiotics may play a key role in alleviating exercise-induced fatigue in mice.

Growing evidence shows that gut microbiota affects system metabolism by altering the host metabolome [51]. In this study, we found that metabolic pathways such as pyruvate metabolism, TCA cycle and amino acid metabolism are closely associated with fatigue, and have a positive response to the therapeutic intervention of FDB. Pyruvate, which is related to energy metabolism, is the starting point of the TCA cycle, and 2-oxoglutarate and citrate are intermediate metabolites of the TCA cycle. The levels of pyruvate, 2-oxoglutarate, and citrate were significantly increased in the urine of FDB-treated mice, indicating a potentially beneficial role of FDB in TCA cycle and energy metabolism. Creatine phosphate is formed from creatine with ADP to produce ATP and creatinine, which provides energy for muscles and nerve cells [52]. In this study, the level of creatinine was decreased after intense exercise, indicating the creatine conservation and muscle injury. In FDB-treated groups, the level of creatinine was elevated, demonstrating that FDB might alleviate fatigue by protecting the muscle. Further evidences for the association of disturbance to gut microbiota with exercise-induced fatigue were the altered levels of gut microbial cometabolites including trimethylamine (TMA) and PAG. Previous studies have demonstrated that urinary TMA is produced through the action of gut microbiota on choline [53]. Reduced TMA means that more choline can be used to produce phosphate choline and creatinine. Phenylalanine is converted into phenylacetate under the action of gut microbiota. Then phenylacetate was conjugated with glycine to form PAG [54]. This implied that FDB treatment affected the growth or activity of the microbiota, thereby reducing urinary TMA and PAG levels.

After intensive exercise, the enhancement of protein breakdown compensates the lack of energy supply. Alanine and glycine are the precursor amino acids of gluconeogenesis, and the increase in these two amino acids proves that the protein breakdown level is intensified and the gluconeogenesis level is enhanced. The administrations of FDB significantly affected the levels of alanine and glycine in small intestine of exercise mice. Metabolic disorder of branched chain amino acids (BCAA) affects a variety of diseases [55,56]. BCAAs can be consumed and oxidized in the gut, and participate in bacterial metabolism, regulating the composition and diversity of gut microorganisms [57]. The elevated levels of valine, leucine, and isoleucine in the small intestine of exercise mice were downregulated by FDB administration. This finding indicated that the increase in BCAAs may be due to an increase in proteolysis and a decrease in the diversity of microorganisms using BCAA. Taurine is an abundant amino acid in animal tissues and participates in many important biological processes. It has many beneficial effects such as anti-oxidation, anti-inflammatory, and anti-apoptosis activity [58]. In FDB-treated groups, the level of taurine was restored, which indicated that FDB regulates the taurine and hypotaurine metabolism.

## 5. Conclusions

**Figure 6:**
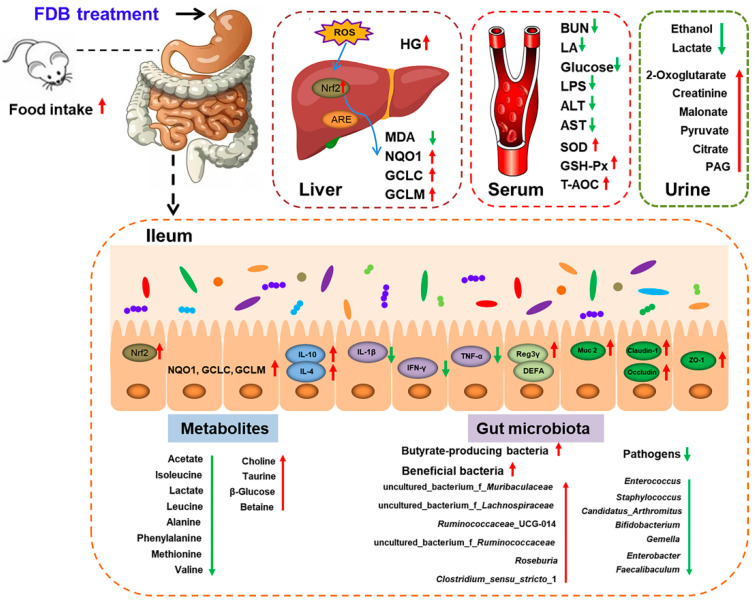
Overview of the effects of FDB on the fatigue related parameters, small intestine microbiota and metabolites in intense exercise mice. BUN: urea nitrogen; LA: lactic acid; LPS: lipopolysaccharide; ALT: alanine aminotransferase; AST: aspartate aminotransferase; HG: hepatic glycogen; SOD: superoxide dismutase; GSH-Px: glutathione peroxidase; T-AOC: total antioxidative capacity; MDA: malondialdehyde; PAG: phenylacetyl glycine.

In conclusion, as shown in Figure 6, the present study demonstrated that FDB can alleviate intense exercise-induced fatigue through several targets including eliminating harmful serum metabolites, increasing glycogen accumulation, reducing oxidative damage, and maintaining intestinal barrier function. Additionally, FDB had the potential to relieve fatigue by modulating the small intestine microbiota and metabolism. The anti-fatigue effects of FDB were associated with the altered abundance of some butyrate-producing bacteria, probiotics, and pathogens in the small intestine content. This effect was also significantly related to the TCA cycle and amino acid metabolism in urine and small intestine content. The comprehensive analysis indicated that FDB may be a potential functional product to improve exercise-induced fatigue and maintain intestinal health.

## Data Availability

All data presented in this study are available on request from the corresponding authors. The data are not be uploaded to a publicly accessible database.

## Supplementary Materials

The following are available online at https://www.mdpi.com/article/10.3390/nu13051543/s1, Table S1: Sequence of primers used for the qRT-PCR assays; Table S2: The chemical composition and antioxidant capacity in DB and FDB; Table S3: Free amino acid composition in DB and FDB; Figure S1: Schematic representation of deer blood fermentation; Figure S2: The schematic diagram of animal experiment; Figure S3: FDB modulates the small intestinal microbiome in fatigued mice; Figure S4: FDB modulates the cecum microbiomes in fatigued mice; Figure S5: Metabolic profile and multivariate analysis in fatigued mice; Figure S6: Metabolic profile and multivariate analysis of cecal content in fatigued mice.
