# Supplementary material for: Fermented Deer Blood Ameliorates Intense Exercise-Induced Fatigue via Modulating Small Intestine Microbiota and Metabolites in Mice
Source: Nutrients. 2021 May 3;13(5):1543. doi: 10.3390/nu13051543 (PMC8147844; doi:10.3390/nu13051543)
Supplement: Supplementary file 1 [file nutrients-13-01543-s001.zip › nutrients-1176325-supplementary.pdf]

Supplementary Materials

Table S1. Sequence of primers used for the qRT-PCR assays.

Genes	Forward primer (5'-3')	Reverse primer (5'-3')
GAPDH	5'-GTGTTCCTACCCCCAATGTGT-3'	5'-ATTGTCATACCAGGAAATGAGCTT-3'
28S	5'-TTGAAAATCCGGGGGAGAG-3'	5'-ACATTGTTCCAACATGCCAG-3'
Nrf2	5'-GTCTTCACTGCCCCTCATC-3'	5'-TCGGGAATGGAAAATAGCTCC-3'
NQO1	5'-ACCTTGCTTTCTATCACCACTG-3'	5'-TGAATCGGCCAGAGAATGAC-3'
GCLC	5'-ACATCTACCACGCAGTCAAGGACC-3'	5'-CTCAAGAACATCGCCTCCATTCAG-3'
GCLM	5'-GCCACCAGATTTGACTGCCTTTG-3'	5'-TGCTCTTCACGATGACCGAGTACC-3'
TNF-α	5'-AGACCCTCACACTCAGATCA-3'	5'-TCTTTGAGATCCATGCCGTTG-3'
IL-1β	5'-TCCATGAGCTTTGTACAAGGA-3'	5'-AGCCCATACTTTAGGAAGACA-3'
IFN-γ	5'-TCAAGTGGCATAGATGTGGAAGAA-3'	5'-TGGCTCTGCAGGATTTTCATG-3'
IL-4	5'-AGATCACGGCATTTTGAACG-3'	5'-TTTGGCACATCCATCTCCG-3'
IL-10	5'-GGACAACATACTGCTAACCGACTC-3'	5'-AAAATCACTCTTCACCTGCTCCAC-3'
Muc2	5'-ACGTGTCATATTTGCACCTCT-3'	5'-TCAACATTGAGAGTGCCAACT-3'
ZO-1	5'-TTTTTGACAGGGGGAGTGG-3'	5'-TGCTGCAGAGGTCAAAGTTCAAG-3'
Occludin	5'-ATGTCCGGCCGATGCTCTC-3'	5'-TTTGGCTGCTCTTGGGTCTGTAT-3'
Claudin-1	5'-CGGGCAGATACAGTGCAAAG-3'	5'-ACTTCATGCCAATGGTGGAC-3'
Reg3γ	5'-TTCCTGTCCTCCATGATCAAA-3'	5'-CATCCACCTCTGTTGGGTTC-3'
DEFA	5'-GGTGATCATCAGACCCCAGCATCAGT-3'	5'-AAGAGACTAAAACTGAGGAGCAGC-3'

Table S2. The chemical composition and antioxidant capacity in DB and FDB.

Project	DB	FDB
Peptide content (mg/g)	0.23 ± 0.09	184.3 ± 3.3
FRAP (mg FE/g)	0.32 ± 0.02	0.76 ± 0.02
DPPH (%)	10.75 ± 0.01	14.86 ± 1.72
Superoxide (%)	ND	41.69 ± 1.77
Hydroxyl (%)	22.03 ± 1.03	62.89 ± 2.75

Data are expressed as mean ± SD (n = 3). DB: non-fermented deer blood;

FDB: fermented deer blood; FRAP: ferric reducing antioxidant power assay; DPPH: DPPH radical scavenging activity; Superoxide: Superoxide radical scavenging activity; Hydroxyl: Hydroxyl radical scavenging activity. FRAP is expressed as mg Fe^{2+} equivalents per g of the sample (mg FE/g sample). ND: not detected.

Table S3. Free amino acid composition in DB and FDB.

Amino acid (mg/g)	DB	FDB
Aspartate	0.025 ± 0.004	3.81 ± 0.15
Threonine	0.033 ± 0.001	1.88 ± 0.02
Serine	0.006 ± 0.000	6.65 ± 0.20
Glutamate	0.046 ± 0.002	5.64 ± 0.33
Proline	0.026 ± 0.001	2.32 ± 0.04
Glycine	0.003 ± 0.000	3.79 ± 0.04
Alanine	0.023 ± 0.002	3.59 ± 0.27
Valine	0.038 ± 0.008	18.24 ± 1.23
Methionine	0.016 ± 0.001	3.90 ± 0.14
Isoleucine	0.021 ± 0.000	2.01 ± 0.07
Leucine	0.020 ± 0.001	31.68 ± 0.05
Tyrosine	0.018 ± 0.001	10.13 ± 0.28
Phenylalanine	0.008 ± 0.001	25.30 ± 1.20
Histidine	0.033 ± 0.001	1.99 ± 0.00
Lysine	0.032 ± 0.002	18.77 ± 0.03
Arginine	0.006 ± 0.000	0.039 ± 0.004
Total	0.35 ± 0.02	139.7 ± 0.5

Data are expressed as mean ± SD (n = 3). DB: non-fermented deer blood; FDB: fermented deer blood.

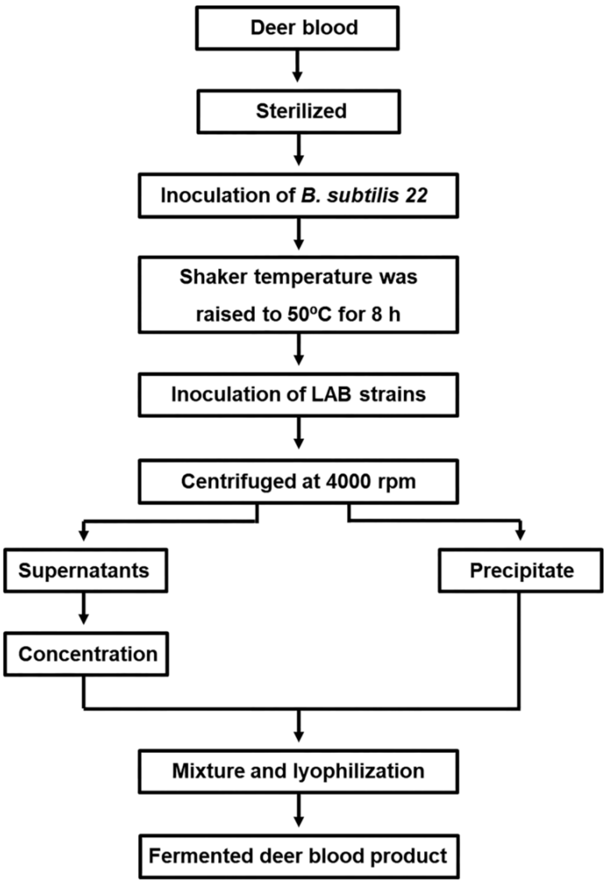

Figure S1. Schematic representation of deer blood fermentation.

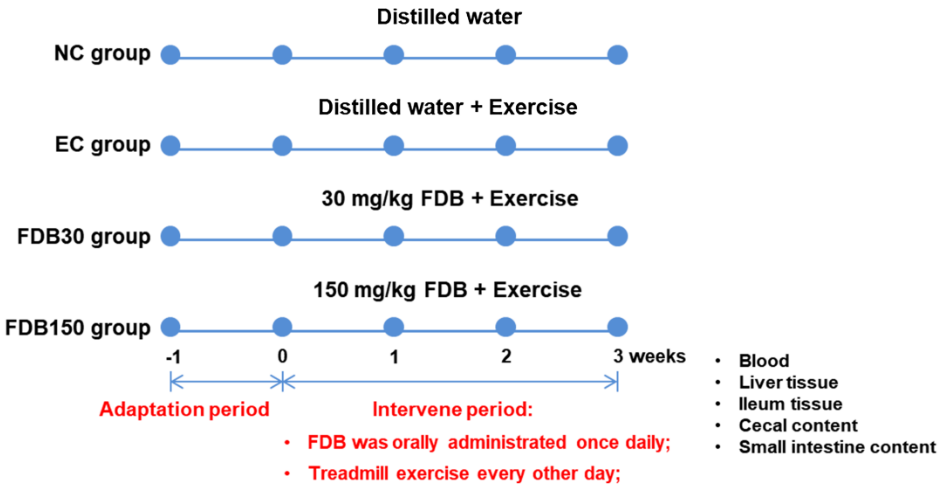

Figure S2. The schematic diagram of animal experiment. FDB: fermented deer blood. Mice were randomly divided into four groups: Normal control (NC); Exercise control (EC); 30 mg/kg FDB (FDB30); 150 mg/kg FDB (FDB150). Each group contains ten mice.

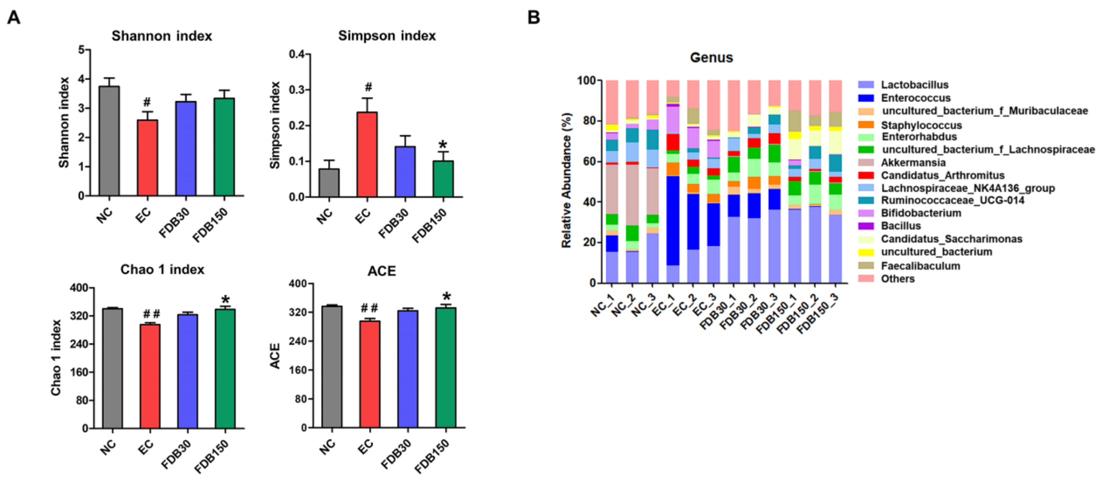

Figure S3. FDB modulates the small intestinal microbiome in fatigued mice. (A) The α diversity indexes of Shannon, Simpson, Chao 1 and ACE. (B) Histogram of microbial composition at the genus level. $^{\#}$ $p < 0.05$, $^{\#\#}$ $p < 0.01$, and $^{\#\#\#}$ $p < 0.001$ vs. NC (normal control) group; * $p < 0.05$, ** $p < 0.01$, and *** $p < 0.001$ vs. EC (exercise control) group. FDB30: 30 mg/kg FDB group; FDB150: 150 mg/kg FDB group.

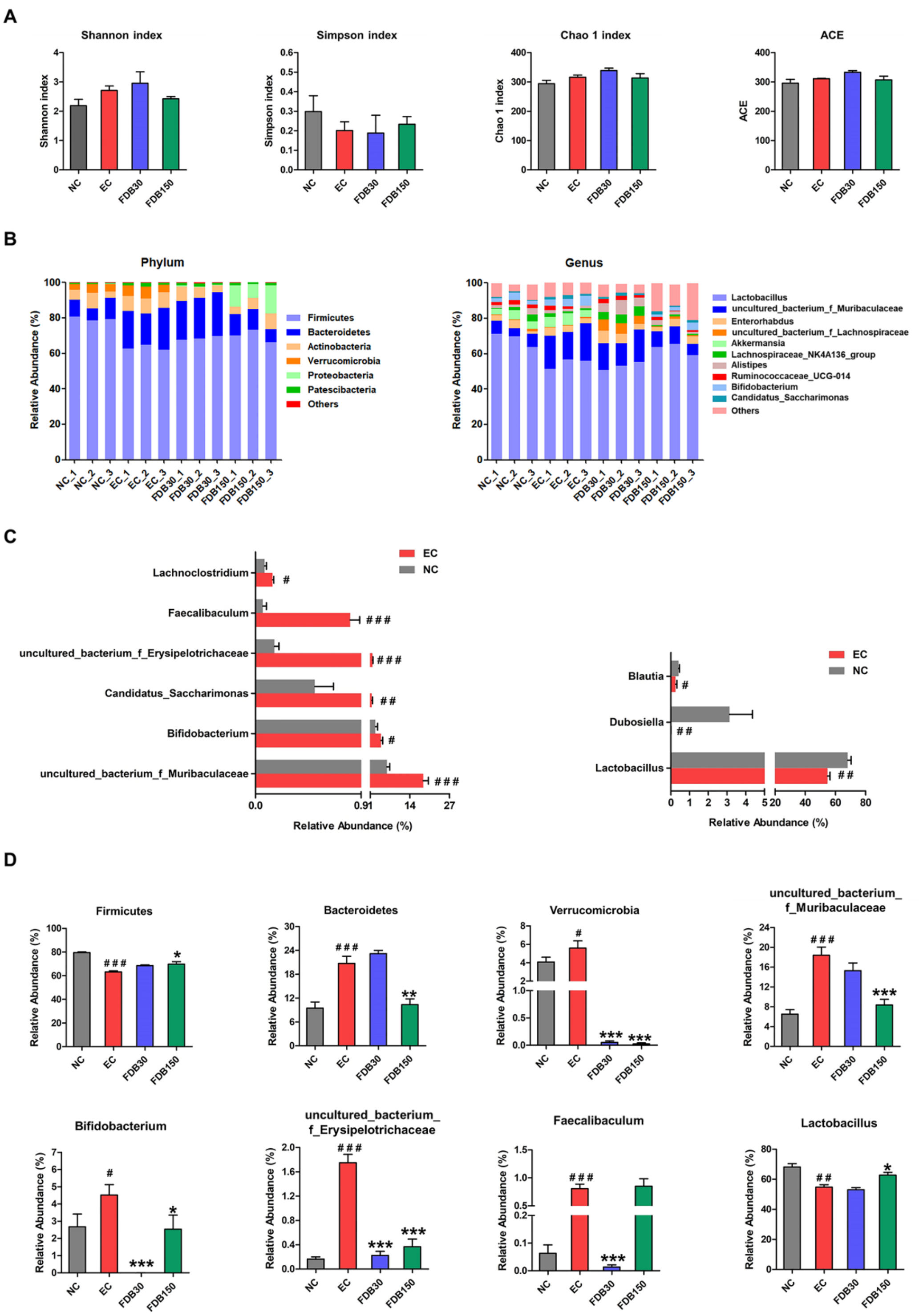

Figure S4. FDB modulates the cecum microbiomes in fatigued mice. (A) The α diversity indexes of Shannon, Simpson, Chao 1 and ACE. (B) Histogram of microbial composition at the phylum and genus levels. (C) Relative abundance of significantly altered bacterial taxa at genus level between NC (normal control) and EC (exercise control) mice. (D) Relative abundance of significantly altered bacterial taxa including phylum and genus levels between EC and FDB mice. $^{\#}p < 0.05$, $^{\#\#}p < 0.01$, and $^{\#\#\#}p < 0.001$ vs. NC (normal control) group; $^{*}p < 0.05$, $^{**}p < 0.01$, and $^{***}p < 0.001$ vs. EC (exercise control) group. FDB30: 30 mg/kg FDB group; FDB150: 150 mg/kg FDB group.

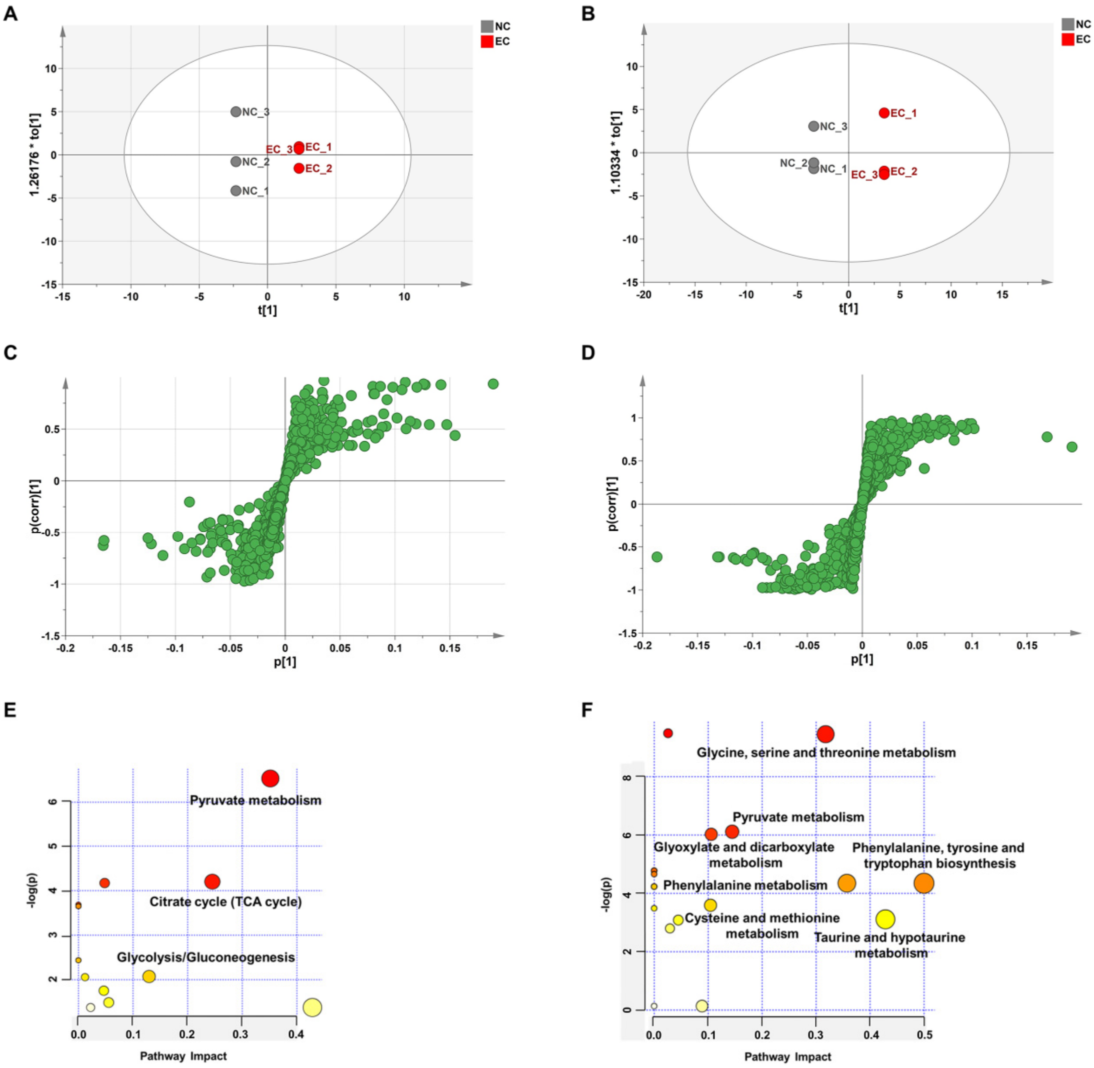

Figure S5. Metabolic profile and multivariate analysis in fatigued mice. OPLS-DA plots with the scores of the first two principal components of (A) urinary and (B) small intestine content samples. S-plots of OPLS-DA model about (C) urinary and (D) small intestine content samples from the NC (normal control) and EC (exercise control) mice. Summary of pathway analysis in (E) urinary and (F) small intestine metabolites.

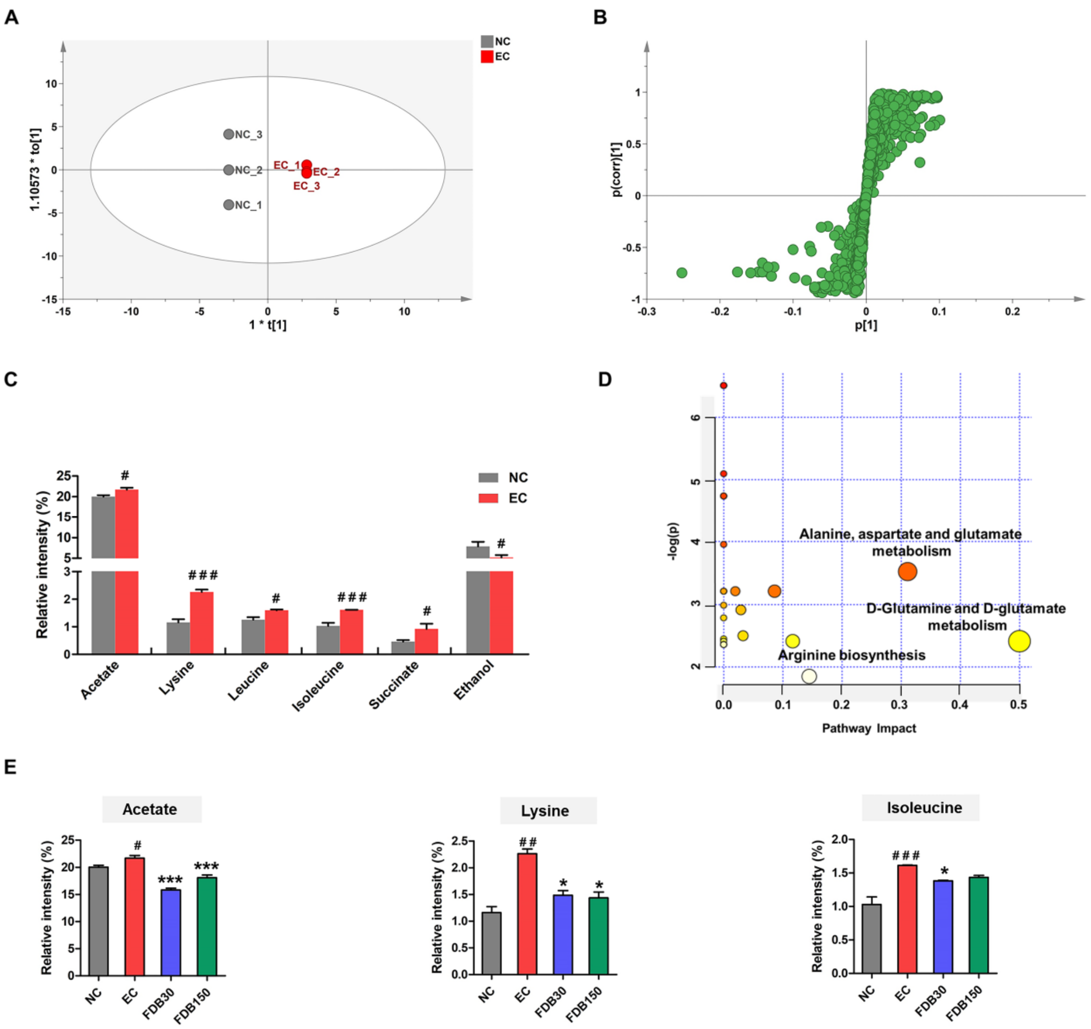

Figure S6. Metabolic profile and multivariate analysis of cecal content in fatigued mice. (A) OPLS-DA plots with the scores of the first two principal components of cecal content sample. (B) S-plots of OPLS-DA model cecal content sample from the NC (normal control) group and EC (exercise control) group. (C) Significant changes in cecal metabolites in NC and EC groups are shown in the histogram. (D) Summary of pathway analysis in cecal metabolites. (E) Significant changes in cecal metabolites in EC and FDB groups are shown in the histogram. $^{\#}$ $p < 0.05$, $^{\#\#}$ $p < 0.01$, and $^{\#\#\#}$ $p < 0.001$ vs. NC (normal control) group; * $p < 0.05$, ** $p < 0.01$, and *** $p < 0.001$ vs. EC (exercise control) group. FDB30: 30 mg/kg FDB group; FDB150: 150 mg/kg FDB group.
